# Systematic review of the firefly genus *Scissicauda* (Coleoptera, Lampyridae, Amydetinae) from Brazil

**DOI:** 10.3897/zookeys.558.6040

**Published:** 2016-02-01

**Authors:** Luiz Felipe Lima Da Silveira, José Ricardo M. Mermudes, Milada Bocakova

**Affiliations:** 1 Programa de Pós-Graduação em Ecologia/UFRJ. Laboratório de Ecologia de Insetos, Departamento de Ecologia, Instituto de Biologia, Universidade Federal do Rio de Janeiro, A0-113, Bloco A, Av. Carlos Chagas Filho, 373, Cidade Universitária,Ilha do Fundão, Rio de Janeiro - RJ – Brazil; 2 Laboratório de Entomologia, Departamento de Zoologia, Instituto de Biologia, Universidade Federal do Rio de Janeiro, A1-107, Bloco A, Av. Carlos Chagas Filho, 373, Cidade Universitária,Ilha do Fundão, Rio de Janeiro - RJ – Brazil; 3 Department of Biology, Faculty of Education, Palacky University, Zizkovo nam. 5, CZ-77140 Olomouc, Czech Republic; 4 Department of Zoology, Faculty of Sciences, Palacky University, tr. 17. listopadu 50, CZ-77146 Olomouc, Czech Republic

**Keywords:** Amydetini, Neotropical Region, Psilocladina

## Abstract

The Amydetinae genus *Scissicauda* McDermott, 1964 is reviewed and redescribed. We describe *Scissicauda balena* **sp. n.** from Brazil as new, and provide illustrations of the structural features and a key to species of both sexes.

## Introduction

The subfamily Amydetinae is a little known firefly group distributed predominantly in South America. Molecular data identified Lampyrinae as sister to Amydetinae (Bocakova et al. 2007, Viviani 2011, Amaral et al. 2014), though the circumscription of the subfamily remained unaddressed due to limited taxon sampling of the studies. Phylogenetic relationships of Amydetinae genera sensu McDermott (1966) has not been clarified yet. The monophyly of Amydetinae has been challenged by Jeng (2008, unpublished), whose analyses involved morphological characters, concluding that the subfamily is polyphyletic.

Most Amydetinae share a complex antennal morphology in the males, except some species of *Vesta*, whose antennae are often serrate. Most of the females remain undescribed. McDermott (1966) assigned Amydetinae to subfamily level, keeping its subgroups as subtribes: Amydetina, Vestina and Psilocladina, the latter with five genera including *Scissicauda*. He supposedly retained these subtribes under Amydetini, although not explicitly quoting this tribe in his catalogue (1966). Though such Psilocladina has been challenged (Jeng 2008, unpublished), we refer to McDermott (1966) subdivisions as to the latest comprehensive study.

McDermott (1964) established *Scissicauda* as a replacement name for the monotypic *Schistura* Olivier, 1911 because it was preoccupied by a balitorid fish genus, *Schistura* McClevelland, 1838 (*cf.* McDermott 1966). *Scissicauda* is easily distinguishable from all other lampyrids by the strongly indented pygidium. Currently, only males of the type species, *Scissicauda disjuncta* (E. Olivier, 1896), from Rio de Janeiro, Brazil are known. Here we present a review of the genus, redescribe the type species *Scissicauda disjuncta*, and provide the female description for the first time, together with phenological data for a population in the Serra dos Órgãos Mountain Range (Rio de Janeiro, Brazil). We also propose *Scissicauda balena* sp. n. as new and provide a key to species of the genus.

## Material and methods

The holotype of *Scissicauda disjuncta* was loaned from the Natural History Museum in Paris (MNHN, A. Taghavian). Other specimens were examined in the Museu de Zoologia de São Paulo, São Paulo, Brazil (MZSP, S. Casari) and Museu Nacional do Rio de Janeiro, Rio de Janeiro, Brazil (MNRJ, M. L. Monné). Additional specimens of *Scissicauda disjuncta* were obtained in the Serra dos Órgãos mountain range (Teresópolis municipality, Rio de Janeiro State, Brazil), using monthly sampled Malaise traps (flight interceptor), arranged in seven transects along an elevation gradient in 850–2030m, separated by approximately 200m distance. Totally, 84 Malaise traps were installed there and operated for a one year period (06/2013-06/2014). Specimens were stored in 92% ethanol and are housed at Coleçao José Alfredo Pinheiro Dutra, Universidae Federal do Rio de Janeiro (DZRJ, J. R. Mermudes). The specimens of *Scissicauda balena* sp. n. were loaned from The Natural History Museum, London (BMNH, M. Geiser).

Terms for structural features follow Jeng et al. (2011), Zaragoza (1995) and Silveira and Mermudes (2013, 2014a, 2014b); Crowson (1938, 1944) for metendosternite nomenclature; and Kazantsev and Perez-Gelabert (2008) for female genitalia. For taxonomic treatment we follow McDermott (1966), which is the most recent species catalogue of Lampyridae. Specimens had the abdomen dissected and boiled in 10% KOH. This clearing procedure was also applied to two entire specimens of the type species. The morphology was examined using a stereomicroscope and photographs were made with the Leica Application Suite CV3 Auto-montage Software.

## Taxonomy

### Amydetinae Olivier, 1907
Psilocladina McDermott, 1964

#### Scissicauda

Taxon classificationAnimaliaColeopteraLampyridae

McDermott, 1964

Scissicauda McDermott, 1964: 10, 39; 1966: 87.
Schistura Olivier, 1911:51 (*nec Schistura* McClevelland, 1838 Actinopterygii).
Aethra Laporte, 1833 (partim). Olivier *in* Wytsman 1907: 16; Blackwelder 1944: 353.
Lychnuris Motschulsky, 1853 (partim). McDermott 1966 (*quid pro quo*).
Schistura Olivier, 1911: 51; McDermott 1964: 10, 39.

##### Type species.

*Lucidota disjuncta* Olivier, 1896, by monotypy.

##### Diagnosis.

Antenna 11-segmented, compressed, filliform to flabellate, uniramose (while biramose in *Psilocladus* and *Pollaclasis*), with dense, upright bristles, rami atmost twice longer than antennomere body, attached basally (distally in *Ethra*). Antennal sockets large, two thirds of frontal width, close-set, reniform, antennifer process distinct. Occiput as wide as one third head width. Apical maxillary palpomere lanceolate. Apical labial palpomere securiform. Pronotum semilunate, with a marginal row of gross, deep punctures. Abdominal terga with posterior angles progressively produced and acute. Tibial spurs present. Tarsomere I 2× longer than II, II 2× longer than III, III of subequal length as IV. Tarsomere IV bilobed, lobes reaching two thirds of length of tarsomere V. Male sternum IX retracted under VIII. Aedeagus with phallus consisting of a dorsal plate basally fused to parameres, symmetric, projected dorsolaterally toward apex; ventral plate with lateral margins sinuose, weakly sclerotized; parameres symmetric, apically rounded, with a ventrobasal process rudimentary or extended beyond phallus.

##### Redescription.

**Figures 1.**
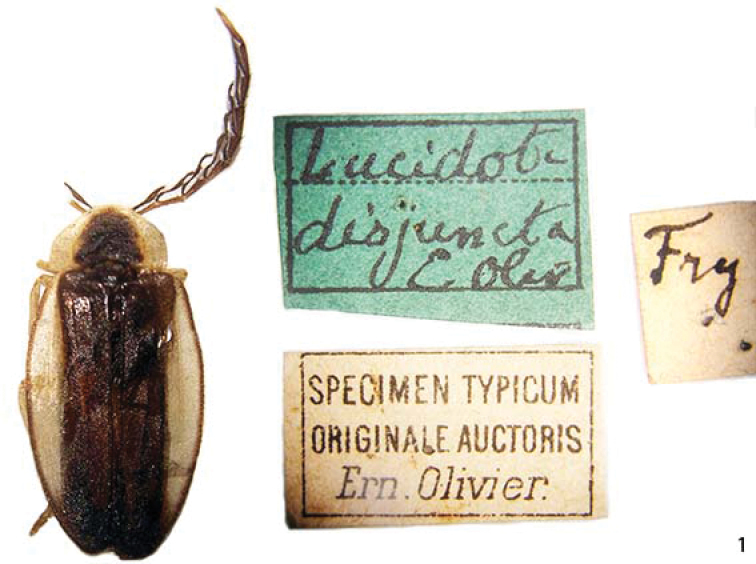
*Scissicauda disjuncta*, holotype and labels.

**Figures 2–3.**
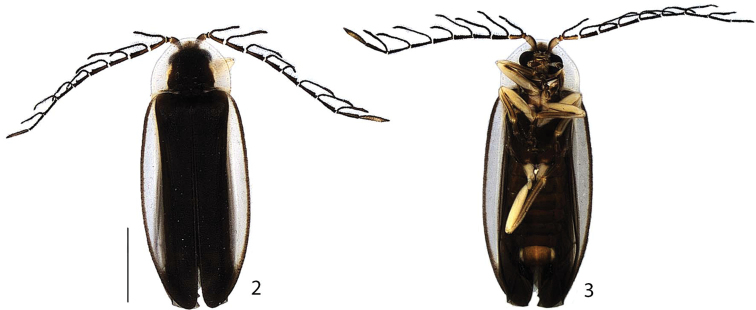
*Scissicauda disjuncta*, male habitus **2** dorsal **3** ventral. Scale bar: 2.0 mm (**2–3**).

**Figures 4–15.**
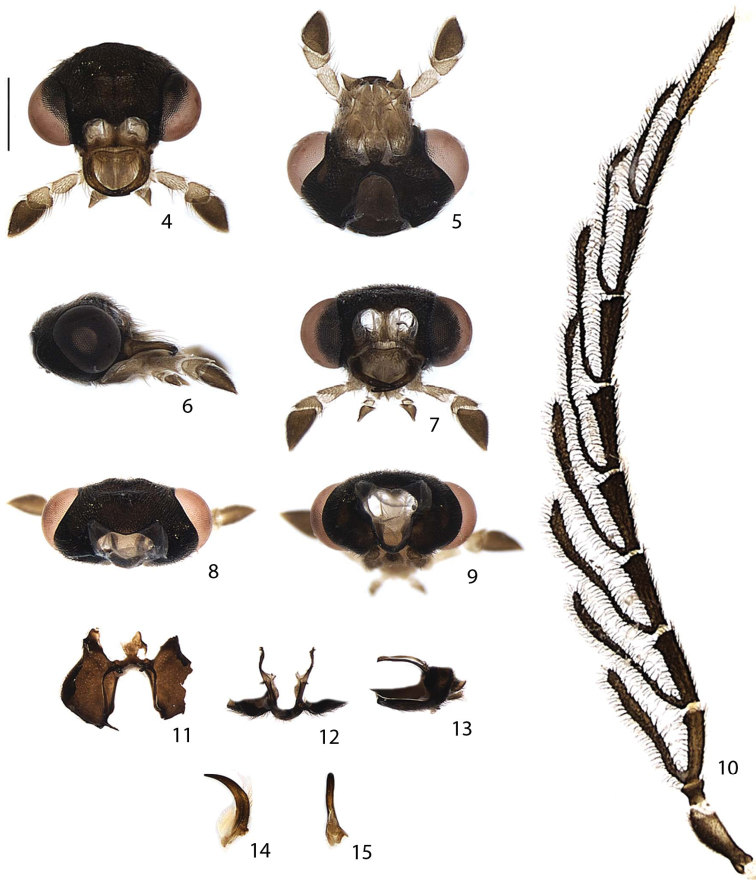
*Scissicauda disjuncta*, male head. **4–9** overview **4** dorsal **5** ventral, **6** lateral **7** frontal **8** posterior **9** occipital **10** antenna, frontal **11–13** tentoria, detail. **11** dorsal **12** frontal **13** lateral **14–15** mandible **14** dorsal **15** internal view. Scale bar: 0.5 mm (**4–15**).

**Head** (Figs 1–15, 44–45, 53–54, 59) entirely covered by pronotum (Figs 1, 2, 42, 51, 67); almost 2× as wide as long, slightly longer than high (Figs 4–7); lateral margins slightly convergent posteriad (Fig. 4). Frons slightly prominent dorsally, swollen (Fig. 6). Antennal sockets reniform, of two thirds frons width; antennifer process conspicuous (Fig. 7). Vertex somewhat convex, with two posterior parasagittal indentations (Fig. 4). Antenna 11-segmented, scape constricted basally, pedicel almost as long as wide and constricted medially, antennomeres III–X serrate to flabellate (males of *Scissicauda disjuncta*), compressed, subequal in length, with dense, upright bristles, lamellae long and slender, subequal in length, apical antennomere slightly longer than subapical one (Figs 10, 42, 54, 68). Frontoclypeus slightly curved (Fig. 7). Labrum connected to frontoclypeus by a membranous suture; 2× as wide as long, anterior margin evanescent (Fig. 4). Mandibles long and slender, monotonically arcuate, apex acute, internal tooth absent, external margin sparsely setose in basal ½, with a basal wisp of bristles up to half its length (Figs 14, 15). Maxilla with cardo well-sclerotized; stipe oblong in ventral view, posterior margins truncate, well-sclerotized, palpi 4-segmented; palpomere III triangular; IV lanceolate, with internal margin covered with minute, dense bristles, almost 3× longer than III (Fig. 7). Labium with mentum well-sclerotized and bristled, completely divided sagittally; submentum sclerotized and bristled, subcordiform, elongate; palpi 3-segmented, palpomere III securiform (Fig. 5). Gular sutures almost indistinct; gular bar transverse, 2× as wide as submentum minimal width. Occiput piriform, as wide as one third posterior width (Fig. 9). Tentorium long and slender, almost as high as half head high, projected internally almost on the half of its length, strongly curved backwards (Figs 11–13).

**Figures 16–20.**
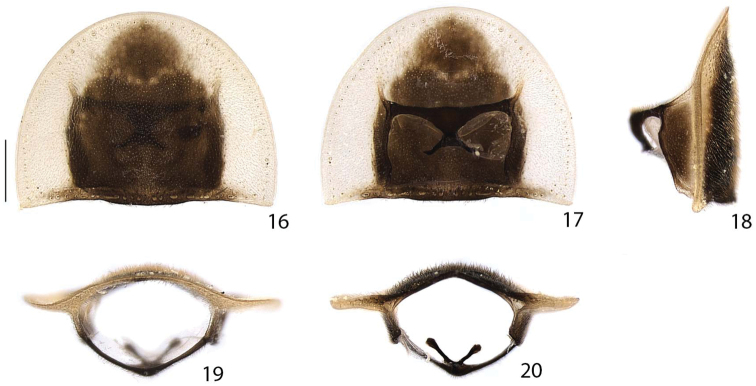
*Scissicauda disjuncta*, prothorax. **16** dorsal **17** ventral **18** lateral **19** frontal **20** posterior. Scale bar: 0.5 mm (**16–20**).

**Figures 21–27.**
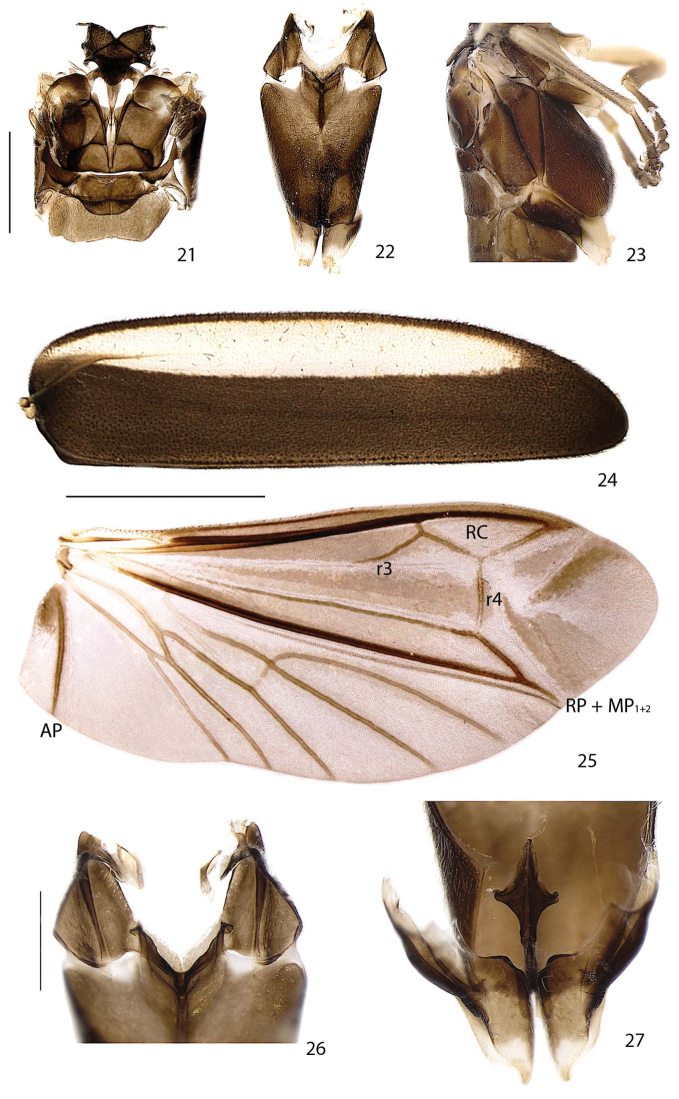
*Scissicauda disjuncta*, pterothorax and associated structures. **21** dorsal **22** ventral **23** lateral **24** elytron, ventral frontal **25** right wing **26** mesoendosternum, posterior **27** metaendosternum, dorsal. Scale bar: 1.0 mm (**21–23**); 2.0 mm (**24–25**); 0.5 mm (**26–27**). **RC** radial cell.

**Figures 28–29.**
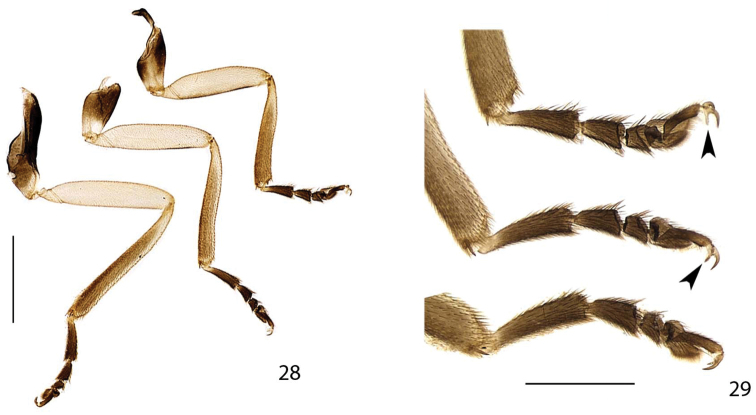
*Scissicauda disjuncta*, male legs. **28** anterior view of right legs **29** detail of tarsus and claw teeth. Scale bar: 1.0 mm (**28**); 0.5 mm (**29**). Arrows: claw teeth.

**Thorax** (Figs 16–29, 46, 55, 56, 70). Pronotum semilunar, posterior angles acute; disc subquadrate in dorsal view, notably convex, regularly punctured, punctures small and bristled; with a line of distinct deep marginal punctures; pronotal expansions well-developed, anterior expansion maximal length almost half as long as disc, posterior expansions straight; slightly wider than humeral distance (Figs 16, 46, 55, 70). Hypomeron longer than high (Figs 18, 56). Prosternum 4× as wide as its major length; slightly constricted parasagitally (Fig. 17). Proendosternite clavate, slightly longer than prosternal process minimal width (Fig. 20). Mesoscutellum with posterior margin rounded (Fig. 21). Elytra ellipsoid, almost 5× as long as wide, pubescent, secondary pubescence absent, with a line of conspicuous punctures all over sutural and lateral margins (Fig. 25).

Hind wing well-developed, posterior margin sinuose, 2× as long as wide, r3 almost as long as r4, radial cell 2× wider than long, almost reaching anterior margin, costal row of setae inconspicuous (Fig. 26); CuA_2_ crossvein absent, mp-cu crossvein present; RP + MP_1+2_ of three fourths r4 length, almost reaching distal margin, J indistinct (Fig. 26). Allinotum slightly wider than long, lateral margins slightly convergent posteriad, posterior margin straight; prescutum extending slightly less than half metascutum length (Fig. 21); rounded area of scutum weakly sclerotized, scutum-prescutal plates sclerotized, extending ridges almost up to posterior margin; metascutellum glabrous. Mesosternum weakly sclerotized, acute medially, attached to metasternum by a suture almost as wide as mesosternum (Fig. 22). Mesoepimeron attached to metasternum by membrane (Fig. 22). Mesosternum/mesanepisternum suture inconspicuous (Fig. 22). Mesanepisternum /mesepimeron suture conspicuous (Fig. 22). Metasternum oblique and strongly depressed by mesocoxae, anterior medial keel prominent up to anterior one third, discrimen indistinct, lateral margins divergent posteriad up to lateral-most part of metacoxa, then convergent posteriad posterior margin bisinuose (Fig. 22). Femur slightly shorter than tibia (Fig. 28). Tibial spurs present (Fig. 28). Tarsomere I 2× longer than II, II 2× longer than III, III subequal in length to IV, IV bilobed, lobes reaching two thirds V length (Fig. 29). Mesendosternum with two parasagittal projections directed outwards, irregularly alate (Fig. 26). Metendosternum spatulate, 2× longer than wide, median projection acute anteriad, with two lateral laminae (Fig. 27).

**Abdomen** (Figs 21, 23, 30–41, 47–50). Tergum I with anterior margin membranous (Fig. 21), laterotergite membranous, polygonal in shape, with sparse bristles (Fig. 23); spiracle obliquely attached to thorax, more vertically (Fig. 21). Terga II–VII with posterior angles progressively produced and acute posteriad, posterior margins progressively bisinuose (Fig. 30). Sterna II–VIII visible (Fig. 31). Spiracles dorsal, at almost half sterna lenghts (Fig. 30). Sternum VIII with larval lanterns elongate (Figs 33–58).

**Figures 30–41.**
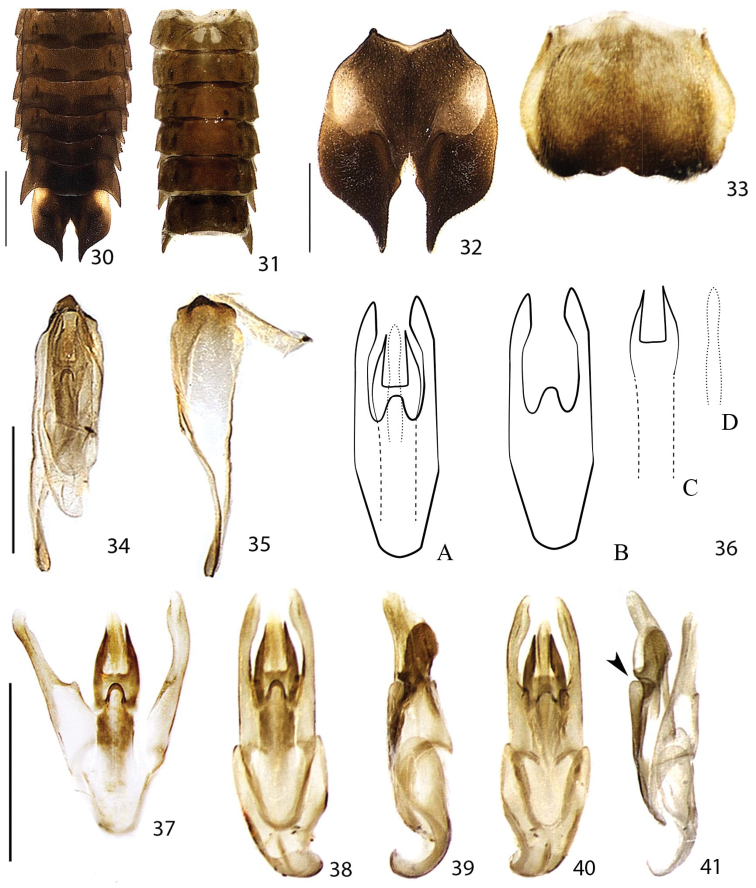
*Scissicauda disjuncta*, male abdomen. **30** dorsal **31** ventral **32** pygidium ventral **33** sternum VIII ventral **34** terminalia, dorsal **35** syntergite and sternum IX, dorsal **36** schematic drawing of aedeagus, dorsal **A** paramerae and phallum **B** paramerae, dashed lines show basal part of dorsal plate **C** Phallic dorsal plate, dashed lines show basal part **D** ventral plate **37** dissected phallum and paramerae, dorsal **38–41** aedeagus **38** dorsal, **39** lateral **40** ventral **41** lateral view, dissected. Scale bar: 1.0 mm (**30–31**); 0.5 mm (**32–33**); 0.5 mm (**34–35**); 0.5 mm (**37–41**). Arrow: phallic groove.

**Male.** Syntergite consisting of paired lateral plates convergent posteriad (putatively tergite IX or paraproct), median transversal suture absent (Figs 34, 35, 61, 62). Sternum IX asymmetric, posterior margin acute. Aedeagus with phallus consisting of a dorsal plate basally fused to parameres, symmetric, medially grooved, projected dorsolaterally toward apex (Figs 36–38, 41, 63, 64); ventral plate with lateral margins sinuose, weakly sclerotized; parameres symmetric, apically rounded, with a ventrobasal process rudimentary or projected and extended beyond phallus (Figs 40, 65, 66).

**Female.** Sternum VIII as long as wide, spiculum ventrale long and slender, three fourths sternum length (Fig. 47). Internal genitalia with a large and somewhat rounded spermatophore-digesting gland anteriad to the common oviduct (Fig. 50). Valvifers free, twisted basally, 3× coxite length; coxites medially fused, coxital baculi well-developed, sclerotized, divergent basally; styli minute, sclerotized; proctiger indistinct (Fig. 49).

##### Remarks.

Concerning the etymology for the generic name, McDermott (1964) did not refer explicitly to the meaning of *Scissicauda*, neither did Olivier (1911) for *Schistura*. *Scissi* is putatively derived from the English word scissor, which in turns refer to the old French *cisoires* and the Latin *caedo*, *caesus*; and *cauda*, a Latin word for the pygydium (Brown, 1956) (see Figs 30, 32). *Scissicauda* is of a feminine gender.

##### Key to species (both sexes)

**Table:** 

1	Elytron with sutural margin brown to blackish-brown (Figs 1–2, 42); hypomeron constricted posteriad (Fig. 18); male antennae flabellate (Fig. 10); lateral margins of female terminal sternum convergent posteriad, indented medially (Fig. 43) (BRAZIL: *Rio de Janeiro*)	***Scissicauda disjuncta* (Olivier, 1896)**
1’	Elytron with sutural margin pale yellow (Figs 51, 67); hypomeron rather bisinuose (Fig. 56); male antennae serrate, without branches (Fig. 54); female terminal sternum rounded (Fig. 68) (BRAZIL: *Espírito Santo*)	***Scissicauda balena* sp. n.**

**Figures 42–43.**
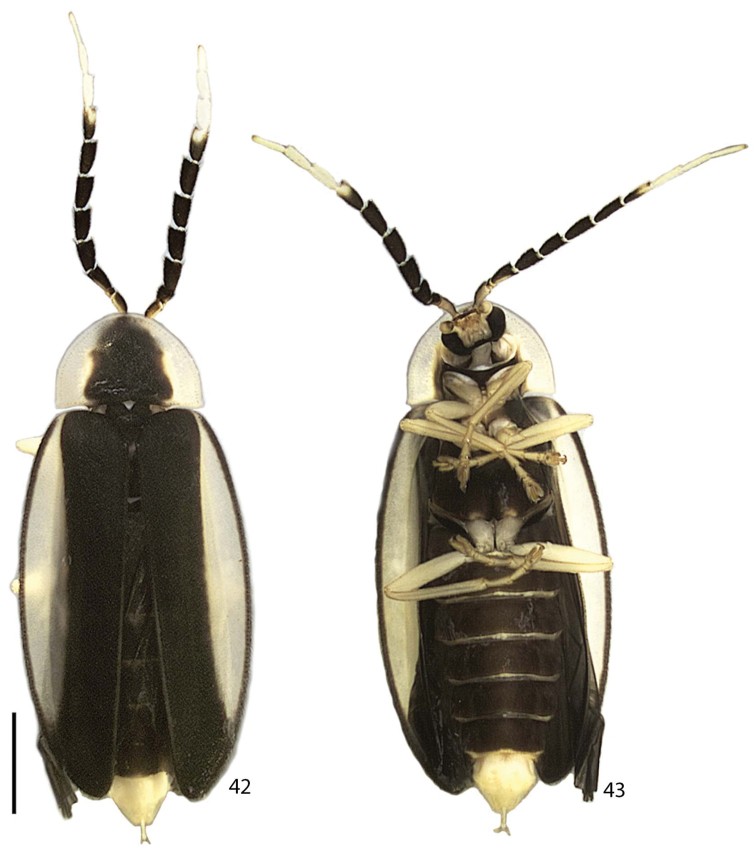
*Scissicauda disjuncta*, female habitus. **42** dorsal **43** ventral. Scale bar: 2.0 mm (**42–43**).

**Figures 44–46.**
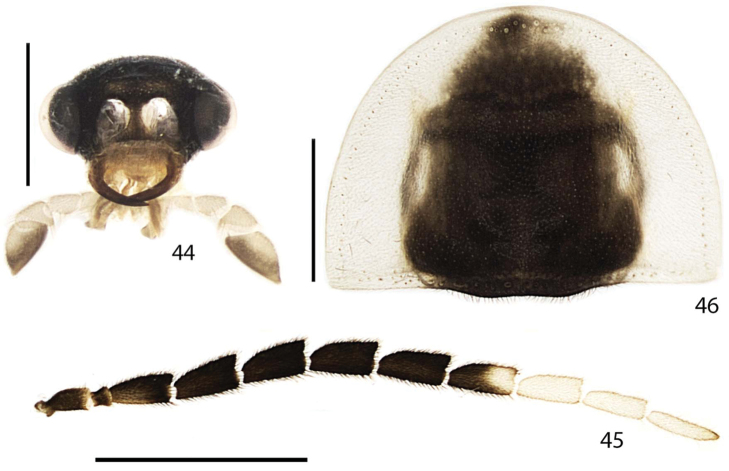
*Scissicauda disjuncta*, female. **44** head, frontal **45** antenna **46** pronotum, dorsal. Scale bar: 1.0 mm (**44**); 2.0 mm (**45**); 1.0 mm (**46**).

**Figures 47–50.**
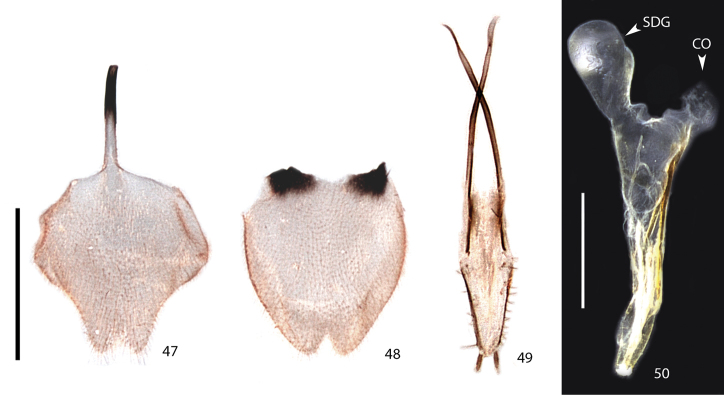
*Scissicauda disjuncta*, female. **47** sternum VIII **48** pygidium, dorsal **49** external genitalia **50** internal genitalia. CO = common oviduct, SDG = spermatophore digesting gland. Scale bar: 1.0 mm (**47–49**); 1.0 mm (**50**).

**Figures 51–56.**
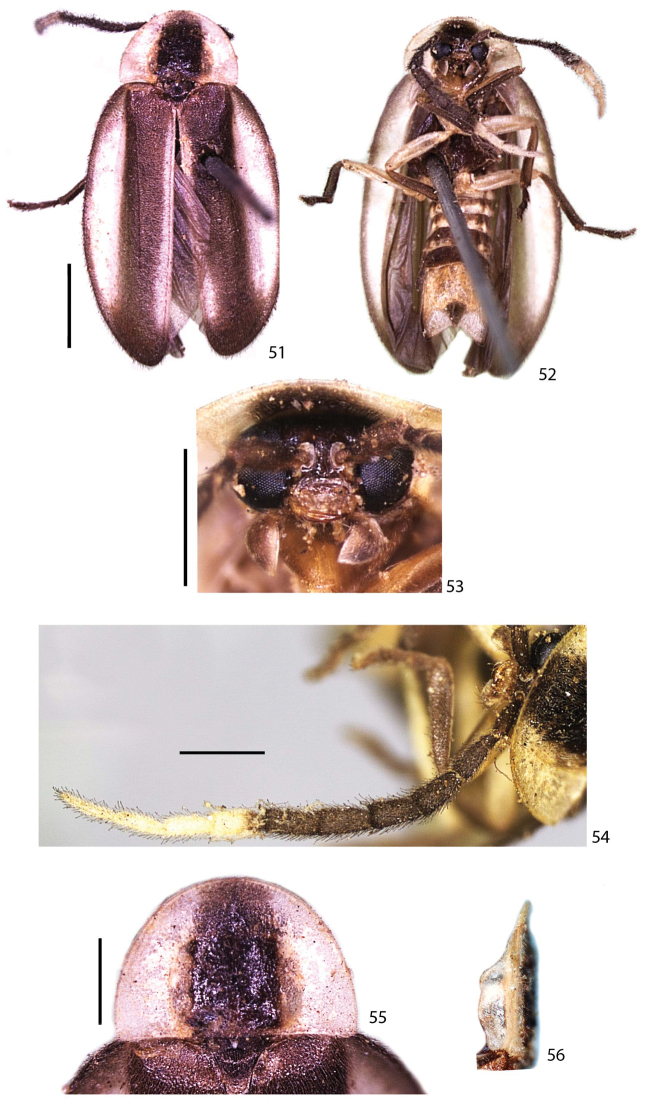
*Scissicauda balena* sp. n., holotype male. **51–52** habitus **51** dorsal **52** ventral **53** head, frontal **54** antenna, dorsal **55–56** prothorax **55** dorsal **56** lateral. Scale bar: 2.0 mm (**51–52**); 1.0 mm (**53**); 1.0 mm (**54**); 1.0 mm (**55–56**).

**Figures 57–66.**
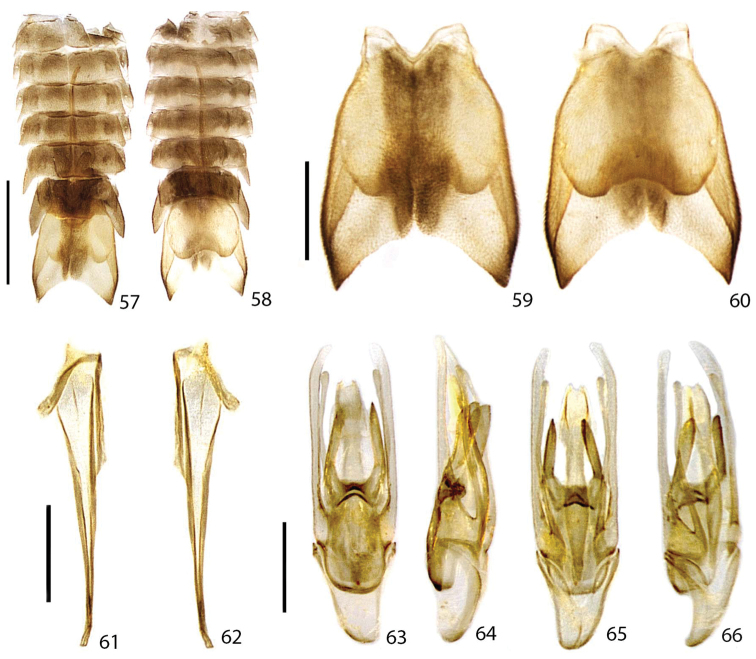
*Scissicauda balena* sp. n., male abdomen. **57–58** abdomen **57** dorsal **58** ventral **59** syntergite dorsal **60** sternum IX ventral. segment VIII **61** pygidium, dorsal **62** sternum VIII, ventral **63–66** aedeagus **63** dorsal **64** lateral **65** ventral **66** oblique. Scale bar: 2.0 mm (**57–58**); 1.0 mm (**59–60**); 1.0 mm (**61–62**); 0.5 mm (**63–66**).

**Figures 67–70.**
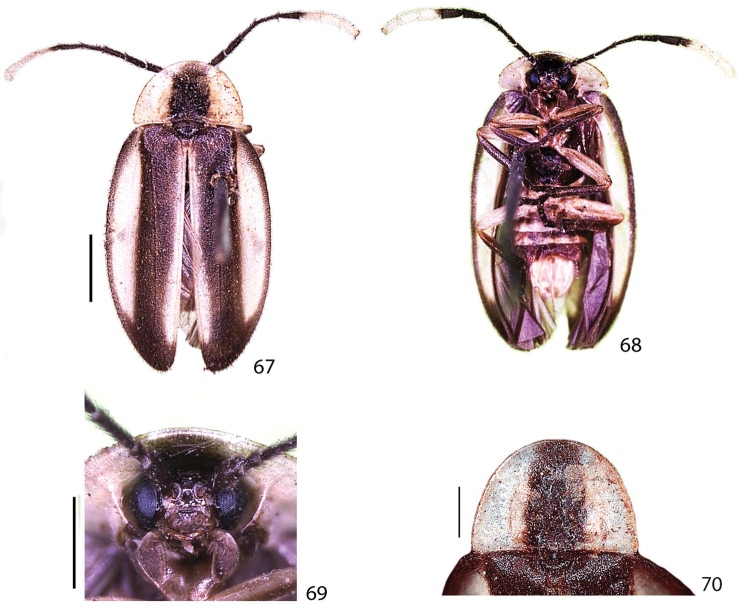
*Scissicauda balena* sp. n., paratype female. **67–68** habitus **67** dorsal **68** ventral **69** head, frontal **70** pronotum dorsal. Scale bar: 2.0 mm (**67–68**); 1.0 mm (**69**); 1.0 mm (**70**).

**Figures 71–72.**
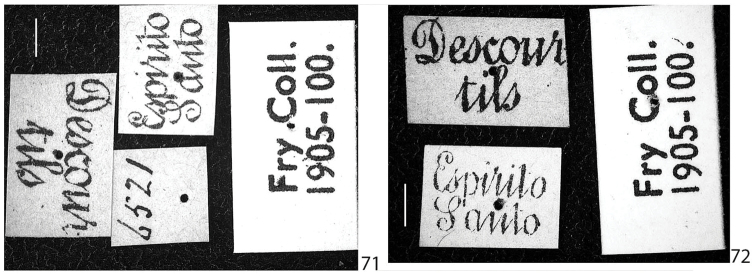
*Scissicauda balena* sp. n., labels. **71** holotype **72** paratype. Scale bar: 2.0 mm (**71–72**).

#### Scissicauda disjuncta

Taxon classificationAnimaliaColeopteraLampyridae

(E. Olivier, 1896)

Figs 1 , 2–3 , 4–15 , 16–20 , 21–27 , 28–29 , 30–41 , 42–43 , 44–46 , 47–50

Lucidota disjuncta Olivier, 1896: 1.
Aethra disjuncta (Olivier, 1896). Olivier *in* Wytsman, 1907: 16; Blackwelder 1944: 353.
Schistura disjuncta (Olivier, 1896). Olivier 1911: 51; McDermott 1964: 10, 39.
Lychnuris disjuncta (Olivier, 1896); McDermott 1966 (*quid pro quo*).
Scissicauda disjuncta (Olivier, 1896). McDermott 1964: 10, 39; 1966: 87.

##### Type material.

**Holotype** (Fig. 1) male (MNHN), without locality data (although Olivier 1911 reported the species from Rio de Janeiro). Bearing the labels: 1) green and rectangular, handwriting *Lucidota disjuncta* E. Oliv.; 2) white and rectangular, printed, *Specimen typicum originale auctoris* Ern. Olivier.; 3) white and square, handwriting, Fry.

##### Material examined.

BRAZIL. *Rio de Janeiro*. Rio de Janeiro, without other data, 1 male, 2 females, Fry coll. (BMNH); Petrópolis, P. N. Serra dos Órgãos, 25/11/2012, Mermudes & Mattos col. (DZRJ); Teresópolis, P. N. Serra dos Órgãos, 15/XII/2014, A. Katz col. (DZRJ), ~1100m, 14-17/I/2015, L. Silveira col. (DZRJ), 18/XII/2014, 1 female, V.A.C WILSON col. (DZRJ), 1050m, XII/2013, Malaise trap, 1 male, 2 females, R. Monteiro col. (DZRJ), 1050m, I/2014, Malaise trap, 2 females, R. Monteiro col. (DZRJ), 1050m, II/2014, Malaise trap, 2 females, R. Monteiro col. (DZRJ).

##### Diagnosis.

Males with antennae flabellate (Fig. 10) (filiform in *Scissicauda balena* sp. n.), anterior pro and mesoclaws bifid (Fig. 29) (entire in *Scissicauda balena* sp. n.), phallus dorsal plate strongly rounded basally, phallic groove at apical one third, strongly curved (subtruncate basally, phallic groove at half its length, moderately curved in *Scissicauda balena* sp. n.); ventral plate at least 2× phallobase length (slightly shorter than phallobase in *Scissicauda balena* sp. n.); parameres ventrobasal process rudimentary (Figs 36–41) (digitiform, extending slightly beyond ventral plate, shorter than paramere itself in *Scissicauda balena* sp. n., Figs 63–66). Female sternum VIII constricted at posterior one third, indented medially (Fig. 43) (rounded in *Scissicauda balena* sp. n., Fig. 68).

##### Description.

**Colour pattern.** Integument from entirely brown to blackish-brown, scape and pedicel yellowish-brown (Figs 1, 2), legs with trochanters, femora and tibial base yellowish, tibiae progressively darkening toward apex (Fig. 3). Prothorax with translucent to slightly pale yellow peripheral semicircular margin, sometimes bearing orangish vittae (Fig. 2), hypomeron antero-dorsally yellowish (Fig. 18). Elytra with pale yellow lateral-longitudinal vittae (Figs 1–3, 24), sutural margin and outer lateral line brown to blackish-brown. Sternum VII with lateral margins yellowish (Fig. 33). Pygidium with anterior angles yellowish (Figs 30, 32).

**Male.** Antennae (Fig. 10) with scape constricted basally, pedicel almost as long as wide and constricted medially; antennomeres III–X subequal in length, slightly serrate and basally flabellate, lamellae almost 2× as long as antennomeres, except for branch X, which is one third longer than antennomere; antennomere XI filiform, slightly longer than previous one. Pronotum 1.3× wider than long (Figs 1–3, 16–18). Abdominal sternum II with two median close-set vitreous spots (Fig. 31), sternum VIII with posterior margin trisinuose (Fig. 33). Sternum IX abruptly constricted anteriad at half its length, one third longer than aedeagus (Figs 34–35). Phallus dorsal plate strongly rounded basally, phallic groove at apical one third, strongly curved; ventral plate slightly shorter than phallobase; parameres ventrobasal process rudimentary (Figs 36–41).

**Female.** Antennomeres III–XI compressed, subequal in length, antennomeres III–X serrate (Figs 42–34, 45). Sternum VIII as long as wide (Fig. 43), constricted at posterior one third, indented medially. Spiculum ventrale long and slender, three fourths sternum length. Sclerotized part of internal genitalia with a large and somewhat rounded spermatophore-digesting gland anteriad to common oviduct. Bursa plate and median oviduct plate absent. Valvifers free, twisted basally, 3× longer than coxite; coxites medially fused, coxital baculi well-developed, sclerotized, divergent basally; styli minute, sclerotized; proctiger indistinct (Figs 47–50) .

**Biology.** Active during daytime, on moisty days. In our experimental design (Jun/2013–Jun/2014), individuals were only collected between December and February, when there is a local increase in pluviosity (Graphic 1). Our results suggest that *Scissicauda disjuncta* breeds during the rainy seasons, possibly in low montane forests. Otherwise, although it could in principle be a sampling artifact, it could also mean that the species has a patchy distribution.

**Graphic 1.**
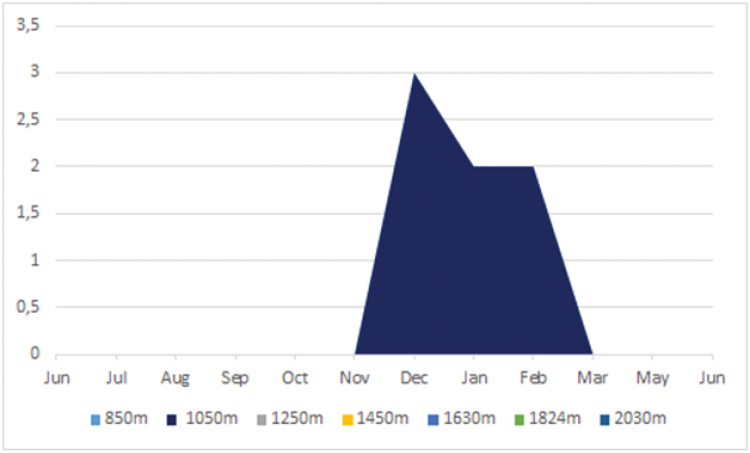
For the period of Jun/2013–Jun/2014, *Scissicauda disjuncta* was sampled at 1250m of elevation and had an abundance peak in the rainy season, between the November–February in the Serra dos Órgãos mountain range.

##### Remarks.

McDermott (1966:87) quoted *Lychnuris disjuncta* referring it to Olivier 1899: 91, but in this paper there is no reference to such a name. However, on page 90, there is a *Lychnuris adjuncta* Olivier, 1899, which is not quoted under *Lychnuris* in his catalogue (McDermott 1966). Therefore we consider the citation a *quid pro quo*. Regarding the etymology of the specific name, the author did not mention a meaning for *disjuncta*, which is a Latin expression for apart, separate. We tentatively associate it with the separated corners of the pygidium.

#### Scissicauda balena

Taxon classificationAnimaliaColeopteraLampyridae

Silveira, Mermudes & Bocakova sp. n.

http://zoobank.org/3626185C-4C9A-49AD-B2C2-B079D85D7927

Figs 51–56 , 57–66 , 67–70 , 71–72

##### Type material.

**Holotype** (Figs 51–66, 71) male, Brazil: Espírito Santo, [n] 6521, Descourtils [leg.], coll. Fry 1905-100 (BMNH). **Paratype** (Figs 67–70, 72) female, Brazil, the same data (BMNH).

##### Diagnosis.

Males with antennal lamellae absent (Fig. 54) (present in *Scissicauda disjuncta*, Fig. 10), anterior pro and mesoclaws entire (bifid in *Scissicauda disjuncta*), phallus dorsal plate subtruncate basally, phallic groove at half of its length, moderately curved (strongly rounded basally, phallic groove at apical one third, strongly curved in *Scissicauda disjuncta*); ventral plate at least 2× phallobase length (slightly shorter than phallobase in *Scissicauda disjuncta*); parameres ventrobasal process digitiform, extending slightly beyond ventral plate, shorter than paramere itself (Figs 63–66) (process rudimentary in *Scissicauda disjuncta*, Figs 36–41). Females with sternum VIII rounded (Fig. 68) (constricted at posterior one third, indented medially in *Scissicauda disjuncta*, Fig. 43).

##### Etymology.

The specific name *balena* is a Latin expression for whale, whose tail resembles the pygidium of this species. The name is formed as a noun in apposition.

##### Description.

**Colour pattern.** Integument overall blackish-brown, with scape brownish (Fig. 54); antennomeres VIII–XI and sternum VIII entirely yellowish (Figs 52, 68). Pronotum largely yellowish at sides and slenderly anterior at the disc, with paired yellow parasagittal vittae (Figs 55, 70); hypomeron translucent, with antero-dorsal margin yellowish (Fig. 56). Elytron with pale yellow lateral-longitudinal and sutural vittae (Fig. 51, 67). Sternites, trochanters and femorae yellowish, tibiae and tarsi dark-brown (Fig. 52, 68). Abdominal sternites yellowish posteriad (Fig. 52, 68). Pygidium laterally and medially dark-brownish (Fig. 52).

**Male.** (Figs 51–56, 57–66). Scape constricted basally, pedicel almost as long as wide and constricted medially, antennomeres III–X cylindrical, impressed and not-flabellate (Fig. 54). Pronotum 1.5× wider than long (Fig. 55). Elytra with epipleural maximal width as wide as disc width (Fig. 51). Sternum VIII with posterior margin emarginate (Fig. 60). Sternum IX gradually convergent anteriad, almost 2× longer than aedeagus (Figs 61–62). Phallus dorsal plate subtruncate basally, phallic groove at half of its length, moderately curved; ventral plate at least 2× of phallobase length; parameres ventrobasal process digitiform, extending slightly beyond ventral plate, shorter than paramere itself (Figs 63–66).

**Female.** Sternum VIII rounded, indented medially (Fig. 68).

## Discussion

### Systematics

*Scissicauda* has flabellate antennae, mandibles arcuate (“normal mandibles” *auctorum*), elytral secondary pubescence absent, and abdominal spiracles dorsally-oriented, all of which are features of the Amydetinae. The long and diffused antennal branches are features of the Psilocladina. A unique feature amongst the Psilocladina is the abdominal sternum VIII covering sternum IX. However, Psilocladina was deemed polyphyletic on the most comprehensive phylogenetic analysis for the Lampyridae (Jeng 2008).

Phallus with ventral plate is a condition found in other lampyrids as the Luciolinae (Ballantyne et al. 2011), Ototretinae (Janisova and Bocakova 2012), Photurinae (Rosa 2007) and *Amydetes* Illiger, 1807 (Silveira and Mermudes 2014a). However, several other taxa lack it, as Lampyrinae: Lampyrini (Geisthardt, 1982), Cratomorphini and Photinini (Zaragoza, 1995), and Pleotomini (Jeng et al., 2006). Phallus dorsally fused to parameres is a derived condition in the phylogenetic analysis of Jeng (2008) based on morphological characters, the most comprehensive for the lampyrids. This condition is also found in some Lampyrinae, Photurinae and Amydetinae, putatively as an evolutionary convergence, supported by its low consistency index (CI=0,15, Jeng 2008). The fused phallus cannot articulate with the parameres, being thus articulated only with the phallobase.

Finally, *Scissicauda disjuncta* share remarkable similarities on reproductive morphology with some taxa considered basal amongst the Lampyridae (Bocakova et al. 2007, Stanger-Hall et al. 2007), such as: some Photurinae taxa, *e.g. Presbyolampis* spp. (*cf.* Kazantsev and Perez-Gelabert 2008), and *Photuris* (*cf.* Rosa 2007 for male genitalia; L. Silveira dissected some females of this genus); as well as some Luciolinae taxa (reviewed by Ballantyne 1987), especially for the female internal genitalia, that of *Scissicauda disjuncta* being quite similar to *Luciola* Laporte, 1833 (South et al. 2008) and *Aquatica* Fu et al. 2010 (Fu et al. 2012), although lacking bursa and median oviduct plates. Even though its knowledge is still incipient, future phylogenetic evaluation and functional morphology of the firefly female genitalia would certainly enhance lampyrid taxonomy.

### Sexual dimorphism

We describe for the first time the females of *Scissicauda disjuncta* and *Scissicauda balena* sp. n., detailing especially the female internal tract, which is inedit for South American taxa and also for the Psilocladina as a whole. Other psilocladina taxa with known females are *Psilocladus* Blanchard, 1846 and *Pollaclasis* Newman, 1838, both genera showing virtually no secondary sexual dimorphism. In *Scissicauda*, secondary sexual dimorphism is stronger in *Scissicauda disjuncta*, where only the males have long lamellae and teethed pro and mesoclaws. *Scissicauda balena* sp. n. is dimorphic only in abdominal segments VIII and beyond. Besides the slightly greater size of the females in both *Scissicauda* species, there are no other noteworthy dimorphic character.

### Sexual selection and possible function of the pygidium in the genus

We suggest male pygidium is involved in reproduction. This could be either working as a clamp, or by enhancing female fecundity. Clamping structures allow prolonged copulation, which is generally assumed to ensure paternity by preventing other males to access - and thus fertilize the eggs of the female (Wing et al. 1993). The evidence that male pygidium may work as a clamp is that anterior angles of female pygidium, which should attach male abdomen, are sclerotized. Alternatively, male reproductive structures may stimulate females while mating, and thus increase fertilization and/or oviposition rates. It was shown that in polyandric systems, female choice can promote male genitalic diversification (Arnqvist 1998), although the *modus operandi* is still disputed (Hosken and Stockley 2004). Furthermore, structures involved in mating are generally species-specific (which is the case in *Scissicauda*) and evolve fast (Eberhard 2004), often as a consequence of sexual selection (Hosken and Stockley 2004), and may promote reproductive isolation either by structural or sensory lock-and-key, thus avoiding hybridization (Masly 2012). Future field observations and detailed histological studies would be useful to test these hypotheses.

### Endemism

Although similar sampling efforts have been made in other montane areas of the Rio de Janeiro State (notably the Serra da Mantiqueira formation), *Scissicauda* was only collected in the Serra dos Órgãos (Petrópolis and Teresópolis municipality). However, the holotype of *Scissicauda disjuncta* is reported from Rio de Janeiro (Olivier 1896), which could be related to the city or the state (which includes the aforementioned municipalities). Thus, we assume that *Scissicauda disjuncta* is restricted to the Serra dos Órgaos low montane forests, and could have occurred also in the Tijuca Forest, although no specimens collected there were found in any of the entomological collections studied. *Scissicauda balena* sp. n. is described from Espírito Santo State, Brazil, lacking more precise locality data.

## Supplementary Material

XML Treatment for Scissicauda

XML Treatment for Scissicauda disjuncta

XML Treatment for Scissicauda balena
